# Revealing the role of metformin in gastric intestinal metaplasia treatment

**DOI:** 10.3389/fphar.2024.1340309

**Published:** 2024-07-19

**Authors:** Ruoyu Hu, Xia Xue, Xiangdong Sun, Yang Mi, Huijuan Wen, Huayuan Xi, Fuhao Li, Pengyuan Zheng, Simeng Liu

**Affiliations:** ^1^ Henan Key Laboratory of Helicobacter Pylori and Microbiota and Gastrointestinal Cancer, Marshall Medical Research Center, The Fifth Affiliated Hospital of Zhengzhou University, Zhengzhou, China; ^2^ Department of Gastroenterology, The Fifth Affiliated Hospital of Zhengzhou University, Zhengzhou, Henan, China

**Keywords:** network pharmacology, molecular docking, intestinal metaplasia, precancerous diseases, metformin

## Abstract

**Objective:**

Gastric intestinal metaplasia (IM) is a precancerous stage associated with gastric cancer. Despite the observed beneficial effects of metformin on IM, its molecular mechanism remains not fully elucidated. This study aims to reveal the effects and potential mechanisms of metformin in treating IM based on both bioinformatics and *in vivo* investigations.

**Methods:**

The seven public databases (GeneCards, DisGeNET, OMIM, SuperPred, Pharm Mapper, Swiss Target Prediction, TargetNet) were used in this work to identify targeted genes related to intestinal metaplasia (IM) and metformin. The shared targeted genes between metformin and IM were further analyzed by network pharmacology, while the interactions in-between were investigated by molecular docking. In parallel, the therapeutic effect of metformin was evaluated in IM mice model, while the core targets and pathways effected by metformin were verified *in vivo*.

**Results:**

We screened out 1,751 IM-related genes and 318 metformin-targeted genes, 99 common genes identified in between were visualized by constructing the protein-protein interaction (PPI) network. The top ten core targeted genes were *EGFR*, *MMP9*, *HIF1A*, *HSP90AA1*, *SIRT1*, *IL2*, *MAPK8*, *STAT1*, *PIK3CA*, and *ICAM1*. The functional enrichment analysis confirmed that carcinogenesis and HIF-1 signaling pathways were primarily involved in the metformin treatment of IM. Based on molecular docking and dynamics, we found metformin affected the function of its targets by inhibiting receptor binding. Furthermore, metformin administration reduced the progression of IM lesions in Atp4a^−/−^ mice model significantly. Notably, metformin enhanced the expression level of *MUC5AC*, while inhibited the expression level of *CDX2*. Our results also showed that metformin modulated the expression of core targets *in vivo* by reducing the activity of NF-κB and the PI3K/AKT/mTOR/HIF-1α signaling pathway.

**Conclusion:**

This study confirms that metformin improves the efficacy of IM treatment by regulating a complex molecular network. Metformin plays a functional role in inhibiting inflammation/apoptosis-related pathways of further IM progression. Our work provides a molecular foundation for understanding metformin and other guanidine medicines in IM treatment.

## 1 Introduction

Gastric cancer, a prevalent malignant neoplasm affecting the gastrointestinal tract, ranks as the fifth in incidence and third in mortality globally (Sung et al., 2021). Gastric intestinal metaplasia (IM), a type of precancerous condition, is highly associated with gastric cancer (Correa and Piazuelo, 2012). A previous study has found that stem cells play a crucial role in sustaining the equilibrium of the gastric mucosa, and its abnormality can be the primary cause related to IM and gastric cancer (Hayakawa et al., 2021). Due to the monoclonal proliferation of IM cells in human stomach, aberrant stem cells that underwent a specific differentiation may generate IM (Tatematsu et al., 2003). For instance, Mist1 gastric stem cells can result in metaplasia which *Kras* activation initiates (Hayakawa et al., 2015). In Mist1-Kras mice, increased Ras expression in the chief cells can lead to a wide range of metaplastic lineage changes, including IM and spasmolytic polypeptide-expressing metaplasia (SPEM) (Hayakawa et al., 2015). Moreover, blocking the Ras signaling pathway by MEK inhibitor has been reported to reverse gastric precancerous lesions (Choi et al., 2016). A latest evidence suggests that the origin of SPEM may attributed to a subset of chief cells through trans-differentiation, which are developed from de-differentiation from chief cells, and shows more easily induced and expanded by acute parietal cell loss under inflammation (Nam et al., 2010). Chronic inflammation is exacerbated by the loss of parietal cells, resulting in foveolar hyperplasia and SPEM, which may eventually form IM (Giroux and Rustgi, 2017).

Metformin, a commonly prescribed biguanide medication, is commonly used in the treatment of type-2 diabetes (LaMoia and Shulman, 2021). It increases the parietal cell differentiation and inhibits the proliferation of the progenitor cell by regulating *AMPK, KLF4*, and *PGC1α*, which induces gastric acid secretion and prevents gastric cancer development (Miao et al., 2020). Metformin activates AMPK and inhibits mTORC1 pathway to trigger apoptosis and impede cell proliferation/cycle progression in gastric carcinogenesis (Cheung et al., 2022). It significantly decreases the incidence of gastric cancer in *Helicobacter pylori*-eradicated diabetes in a time- and dose-dependent manner, suggesting a potential chemopreventive effect on gastric cancer (Cheung et al., 2019). Although metformin reduces the invasive phenotype and intestinal marker expression in CDX1-overexpressing HFE 145 cells (Choi et al., 2019), the role of metformin in IM treatment has not been well studied.

This study herein tried to obtain metformin-targeted genes and IM-related genes from seven public databases (GeneCards, DisGeNET, OMIM, SuperPred, Pharm Mapper, Swiss Target Prediction, TargetNet). Network pharmacology and molecular docking were performed to screen core genes, and then the interaction between metformin and selected genes was studied. The effects and targets-related mechanisms of metformin were investigated in IM mice model to confirm *in vivo*. Our work aims to preliminarily reveal the mechanism of metformin and its potential in IM therapeutics.

## 2 Materials and methods

### 2.1 Experimental design

**FIGURE 1:**
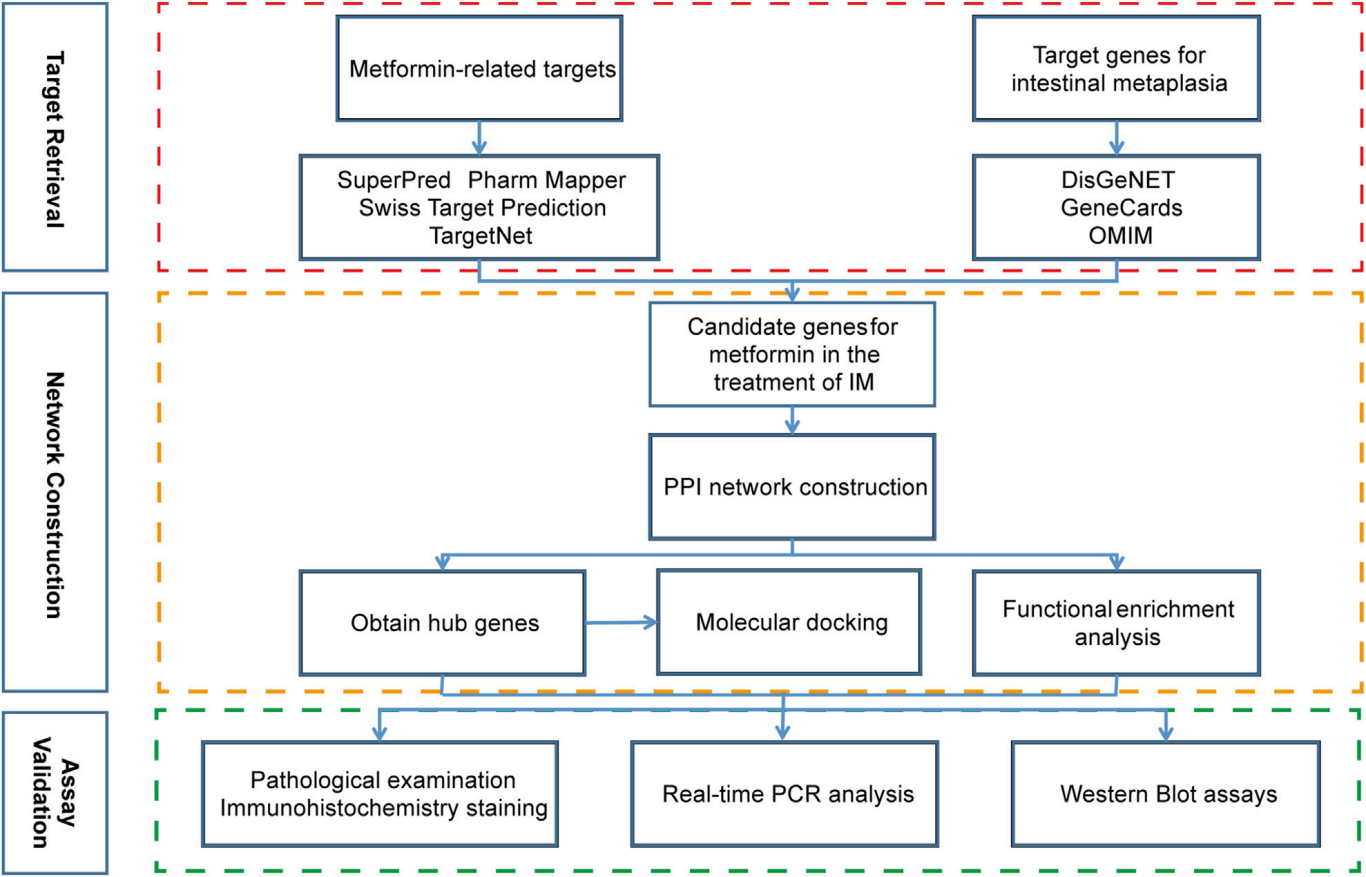
The work flow of this study.

The work flow of this study was shown in Figure 1. The databases and their websites were showed in the Supplementary Table S1.

### 2.2 Identification of IM-related genes

The genes related to IM were obtained from GeneCards (Wang et al., 2022), DisGeNET (He et al., 2022) and Online Mendelian Inheritance in Man (OMIM) (Li et al., 2022) databases. The search term in this work was “intestinal metaplasia.” The Uniprot database (Zhang et al., 2021b) was used for gene correction.

### 2.3 Identification of metformin-targeted genes

Genes targeted by metformin were collected from online databases, including SuperPred (Ma et al., 2022), Pharm Mapper (Jiao et al., 2022), Swiss Target Prediction (Shang et al., 2023), and TargetNet (Zhang et al., 2022c), with “metformin” as the keyword. Uniprot database was used to identify the genes.

### 2.4 Construction and analysis of protein-protein interaction network

With shared targeted genes supplied from IM and metformin collections sourced from the Venny 2.1 tool, the protein-protein interaction (PPI) network was built by the STRING database framework. The visualization of the PPI network was performed using Cytoscape software version 3.9.1 (Zhang et al., 2021a).

### 2.5 Function enrichment analysis

The R clusterProfiler package (Li et al., 2021; Zhou et al., 2022) was conducted for Gene Ontology (GO) and Kyoto Encyclopaedia of Genes and Genomes (KEGG) pathway enrichment analysis. The Bioinformatics website (http://www.bioinformatics.com.cn/) was employed to visualize the enrichment analysis.

### 2.6 Molecular docking

The chemical structure of metformin was obtained from the PubChem database (Zhang et al., 2022b). The PDB database was used to collect the structures of targeted proteins and ligands based on the genes identified above (Nag et al., 2023). The molecular docking study was conducted using AutoDock Vina 1.5.7, and the resulting data was shown using PyMol 2.4.0 (Zhang et al., 2022a; Zhu et al., 2022).

### 2.7 Experimental animals

The Atp4a^−/−^ mice (certificate number: 20170010010146) were constructed utilizing CRISPR/Cas9 system provided by the Shanghai Model Organisms Center. The animals in a pathogen-free room were maintained in standard settings (relative humidity: 50%–60%, temperature: 22°C ± 3°C, 12-h/12-h light/dark cycle). The animal experiments conducted in this study were approved by the Animal Ethics Committee of the Fifth Affiliated Hospital of Zhengzhou University (No. KY2023017). The six wild-type mice (mice-C57bl/6) were purchased from Beijing Weitong Lihua Experimental Animal Technology Company, which were set as the control group, while 12 Atp4a^−/−^ mice were set to the model group and metformin-treated group randomly. Six Atp4a^−/−^ mice in metformin-treated group were given a diet containing 1,000 ppm (0.1%) metformin (Martin-Montalvo et al., 2013; Strong et al., 2016) (Macklin, China), starting at 12 weeks of age. After 12 weeks, gastric tissues were harvested for further experimental analysis.

### 2.8 Pathological examination

The gastric tissue was fixed with 4% paraformaldehyde. The samples were dehydrated by 75%–95% alcohols gradually, immersed in xylene, embedded in paraffin, and then cut into approximately 3-μm sections using the microtome. Haematoxylin and eosin (H&E) staining kit (Celnovte, China) and alcian blue-periodic acid-schiff (AB-PAS) staining kit (pH 2.5) (Celnovte, China) staining were used to observe the degree of gastric lesions.

### 2.9 Immunohistochemistry (IHC) staining

The gastric tissue slices were further dewaxed and hydrated. The gastric tissue sections were incubated with MUC5AC (1:200 dilution; GeneTex, GTX11335, United States) and CDX2 (1:100 dilution; Abcam, ab101532, United Kingdom). The incubation steps were done by the universal two-step kit (mouse/rabbit enhanced polymer test system) (ZSGBBIO, China). The cellular nuclei were stained with haematoxylin (Celnovte, China). The analysis of the integrated optical density (IOD) and area of the images was conducted using the ImageJ software (Schindelin et al., 2012). The protein expression was evaluated using the mean density (IOD/area).

### 2.10 RNA extraction and real-time PCR analysis

Total RNA was extracted from mouse gastric tissues with RNAiso Plus (TAKARA, Japan) following the manufacturer’s procedure. The obtained RNA was reverse-transcribed into cDNA using the ReverTra Ace qPCR RT Kit (Toyobo, Japan). Quantitative real-time polymerase chain reaction (qRT-PCR) was performed with the Roche Lightcycler480II system (Roche, Switzerland) using ChamQ SYBR qPCR Master Mix (Vazyme, China). GAPDH was used as internal control and the qRT-PCR analysis for each sample was performed in triplicate with 2^−ΔΔCT^. The primer sequences were provided in the Supplementary Table S2.

### 2.11 Western blot

The total proteins were extracted from gastric tissues with radio immunoprecipitation assay (RIPA) buffer (Solarbio, Beijing, China) and quantified by Bicinchoninic Acid Assay (Solarbio, Beijing, China). Before sodium dodecyl sulfate polyacrylamide gel electrophoresis (SDS-PAGE), the protein lysis buffer was mixed with 5 × SDS-PAGE loading buffer and boiled for 5 min at 100°C with the thermostat metal bath. The proteins were separated using a 5%–10% SDS-PAGE gel, followed by transferring into a polyvinylidene fluoride (PVDF) membrane. The membranes were blocked using a 5% non-fat milk solution for 2 h at ambient temperature. The membranes were then incubated with the primary antibody at 4°C overnight. After washing three times with Tris-buffered saline with Tween 20 (TBST), the membranes were incubated with the secondary antibodies for 1 h at room temperature. Enhanced chemical luminescence (ECL) reagent (Meilunbio, Shanghai, China) was used to detect the protein bands. The analysis was conducted using the ChemiDocTM XRS + equipment (Bio-Rad, United States).

The information of the primary antibodies are as below: rabbit anti-NF-κB (1:1,000, Cell Signaling Technology, 8,242, United States), rabbit anti-phospho-NF-κB (1:1,000, Cell Signaling Technology, 3,033, United States), rabbit anti-PI3K (1:1,000, Cell Signaling Technology, 4,257, United States), rabbit anti-phospho-PI3K (1:1,000, Affinity Biosciences, AF3241, United States), mouse anti-AKT (1:10,000, Proteintech, 60,203, United States), rabbit anti-phospho-AKT (1:10,000, Proteintech, 80,455, United States), rabbit anti-mTOR (1:5,000, Proteintech, 28,273, United States), rabbit anti-phospho-mTOR (1:1,000, Proteintech, 28,879, United States), rabbit anti-HIF-1α (1:1,000, Cell Signaling Technology, 36,169, United States), and mouse anti-β-Actin (1:10,000, Proteintech, 66,009, United States) (Liu et al., 2020; Ba et al., 2021).

### 2.12 Statistical analysis

GraphPad Prism 9.0 (Berkman et al., 2019) was used to perform all statistical analyses. The Shapiro-Wilk test was used to examine the normal distribution. The independent samples *t*-test was used to compare the two groups of measurement data which met the requirements of normal distribution and homogeneity of variance. One-way analysis of variance (ANOVA) was performed to compare more than two groups of data conforming to the normal distribution and homogeneity of variance requirements. Kruskal–Wallis tests were conducted to compare two groups and multiple groups that did not conform to the normal distribution and homogeneity of variance requirements. The data was shown using the mean ± standard deviation (SD). A *p*-value of <0.05 is indicative of a statistically significant difference.

## 3 Results

### 3.1 Screened IM-related genes and metformin-targeted genes

1,751 IM-related genes were obtained from the DisGeNET, GeneCards, and OMIM databases. In the SuperPred, Pharm Mapper, Swiss Target Prediction, and TargetNet databases, 318 metformin-targeted genes were identified.

### 3.2 GO and pathway enrichment analyses of IM-related genes

**FIGURE 2:**
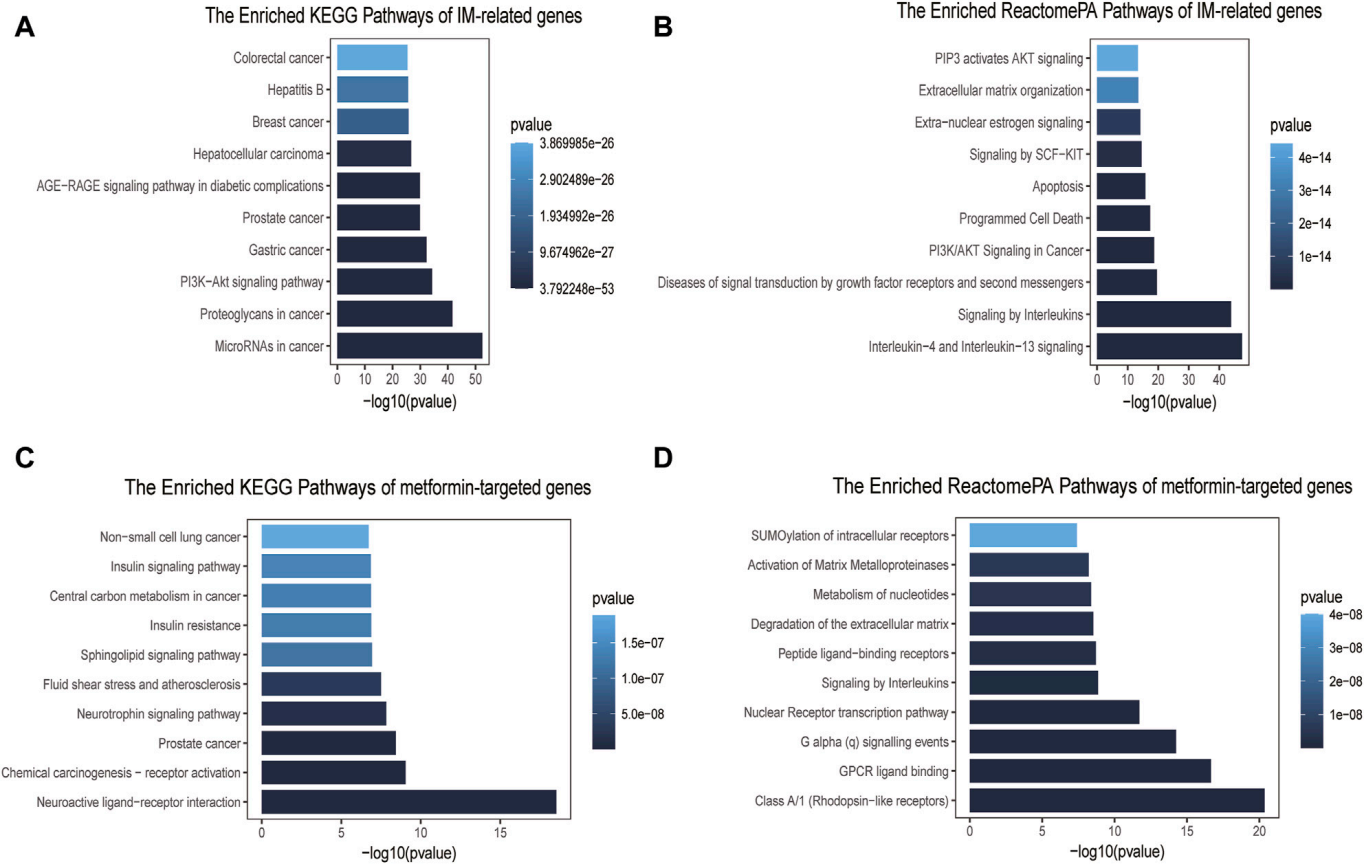
Pathway enrichment analyses of IM-related genes and metformin-targeted genes. **(A)** Top ten enriched KEGG pathways of IM-related genes. **(B)** Top ten enriched ReactomePA pathways of IM-related genes. **(C)** Top ten enriched KEGG pathways of metformin-targeted genes. **(D)** Top ten enriched ReactomePA pathways of metformin-targeted genes.

GO enrichment analysis indicated that cellular components of IM-related genes included collagen−containing extracellular matrix, membrane raft, membrane microdomain, etc (Supplementary Figure S1A). These genes were involved in the activity of receptor ligand, cytokine receptor binding, transcription factor, and growth factor (Supplementary Figure S1B). They were also implicated in several biological processes, such as epithelial cell proliferation, gland development, and tissue migration (Supplementary Figure S1C). We found related signaling pathways, such as microRNAs, proteoglycans, and PI3K-AKT signaling pathway (Figure 2A). Notably, the Enriched ReactomePA Pathways were mainly involved in interleukins related pathways, PI3K/AKT signaling in carcinogenesis and apoptosis (Figure 2B).

### 3.3 GO and pathway enrichment analyses of metformin-targeted genes

The GO enrichment results of metformin-targeted genes showed that the cellular components were mainly related to the vesicle lumen, membrane raft and membrane microdomain (Supplementary Figure S1D), molecular functions were mostly related to the activity of peptide receptor, nuclear receptor and transcription factor (Supplementary Figure S1E), and biological process enrichment items were mainly related to the cellular response to peptide, response to peptide hormone and small molecule catabolic process (Supplementary Figure S1F). The main KEGG pathways were found to be involved in neuroactive ligand-receptor interaction, chemical carcinogenesis-receptor activation, and cancer metabolism (Figure 2C). The ReactomePA pathways enriched analysis primarily included rhodopsin-like receptors, GPCR ligand binding, the nuclear Receptor transcription route, and signaling by Interleukins (Figure 2D).

### 3.4 The analysis of PPI network

**FIGURE 3:**
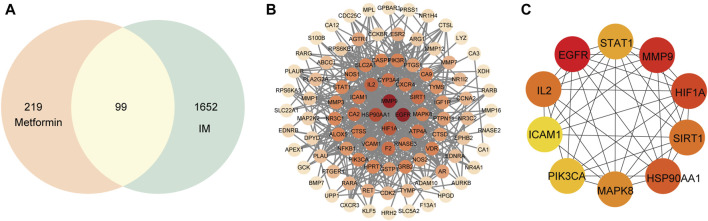
Common targets and PPI network of potential targets. **(A)** Venn diagram of 99 common targets. **(B)** The PPI network was constructed using the STRING and Cytoscape. **(C)** The top ten hub targets were selected from the PPI network.

A total of 99 targets were screened out as the shared targeted genes between IM and metformin clusters (Figure 3A). The PPI network was composed of 94 nodes and 510 edges (Figure 3B). The core targets of metformin in IM treatment were determined by selecting the top ten targets based on their degree value. The PPI network of core targets consists of a total of ten nodes and 41 edges. The top ten targets are *EGFR*, *MMP9*, *HIF1A*, *HSP90AA1*, *SIRT1*, *IL2*, *MAPK8*, *STAT1*, *PIK3CA*, and *ICAM1* (Figure 3C). The shade of the circle represents the importance of the target.

### 3.5 Annotation of targeted genes of metformin in IM treatment

**FIGURE 4:**
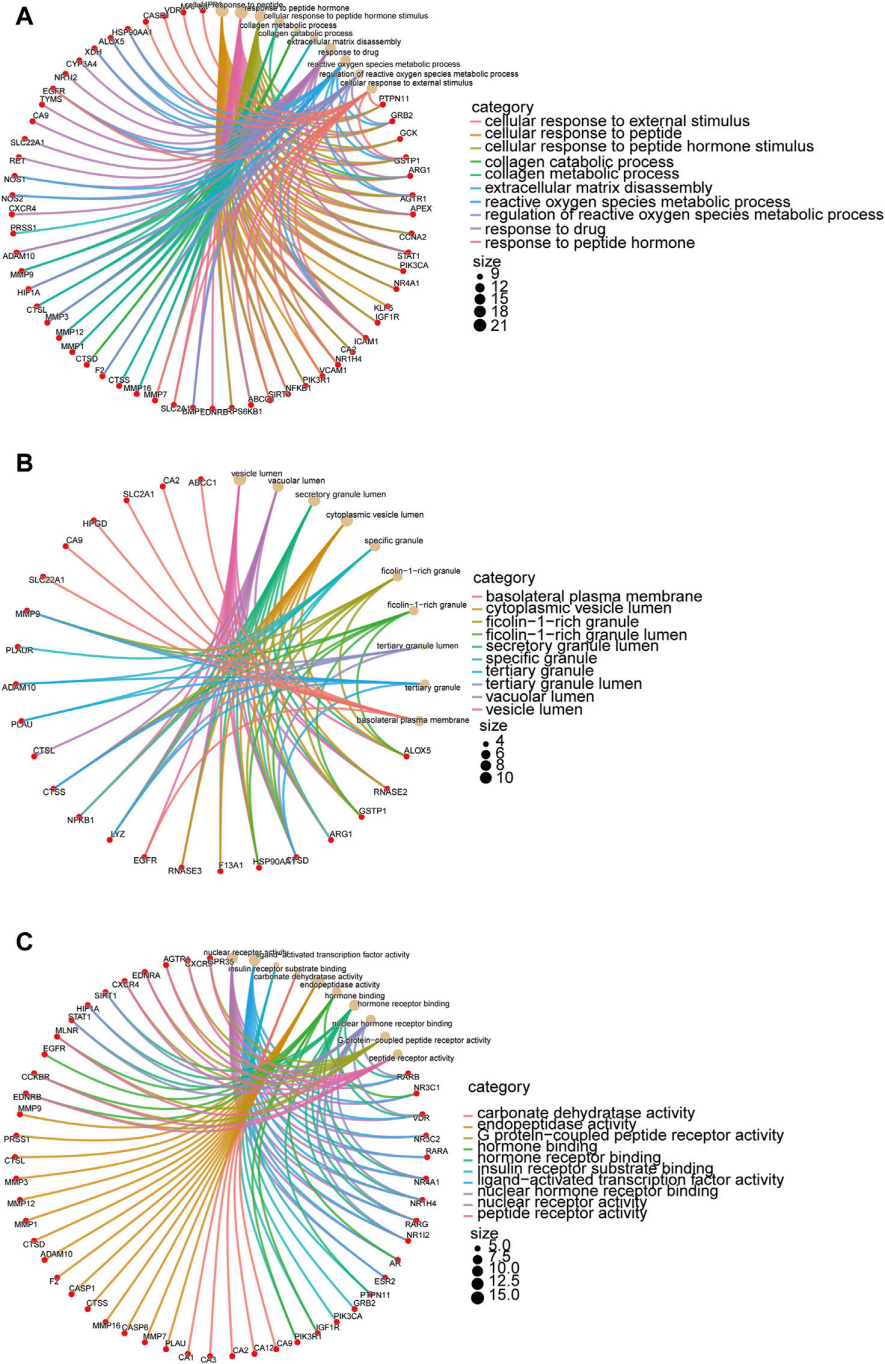
GO enrichment analysis of targeted genes of metformin in IM treatment. The target genes are depicted by red circles, while brown circles represent the results of the enrichment analysis. The interconnecting lines between them refer to mutual relationships. The size of the circle indicates the level of the significance. **(A)** Biological processes of metformin in IM. **(B)** Cellular components of metformin in IM. **(C)** Molecular functions of metformin in IM.

GO and KEGG enrichment analyses were performed on 99 shared targeted genes of metformin for IM treatment. The top ten from each category of GO enrichment analysis were made for visual inspection. Biological processes (Figure 4A) included cellular response to peptide, collagen metabolic process, collagen catabolic process, reactive oxygen species metabolic process, and regulation of reactive oxygen species metabolic process. Cellular components included the vesicle lumen, secretory granule lumen, and specific granule (Figure 4B). The main molecular functions (Figure 4C) were nuclear receptor activity, ligand-activated transcription factor activity, and endopeptidase activity.

**FIGURE 5:**
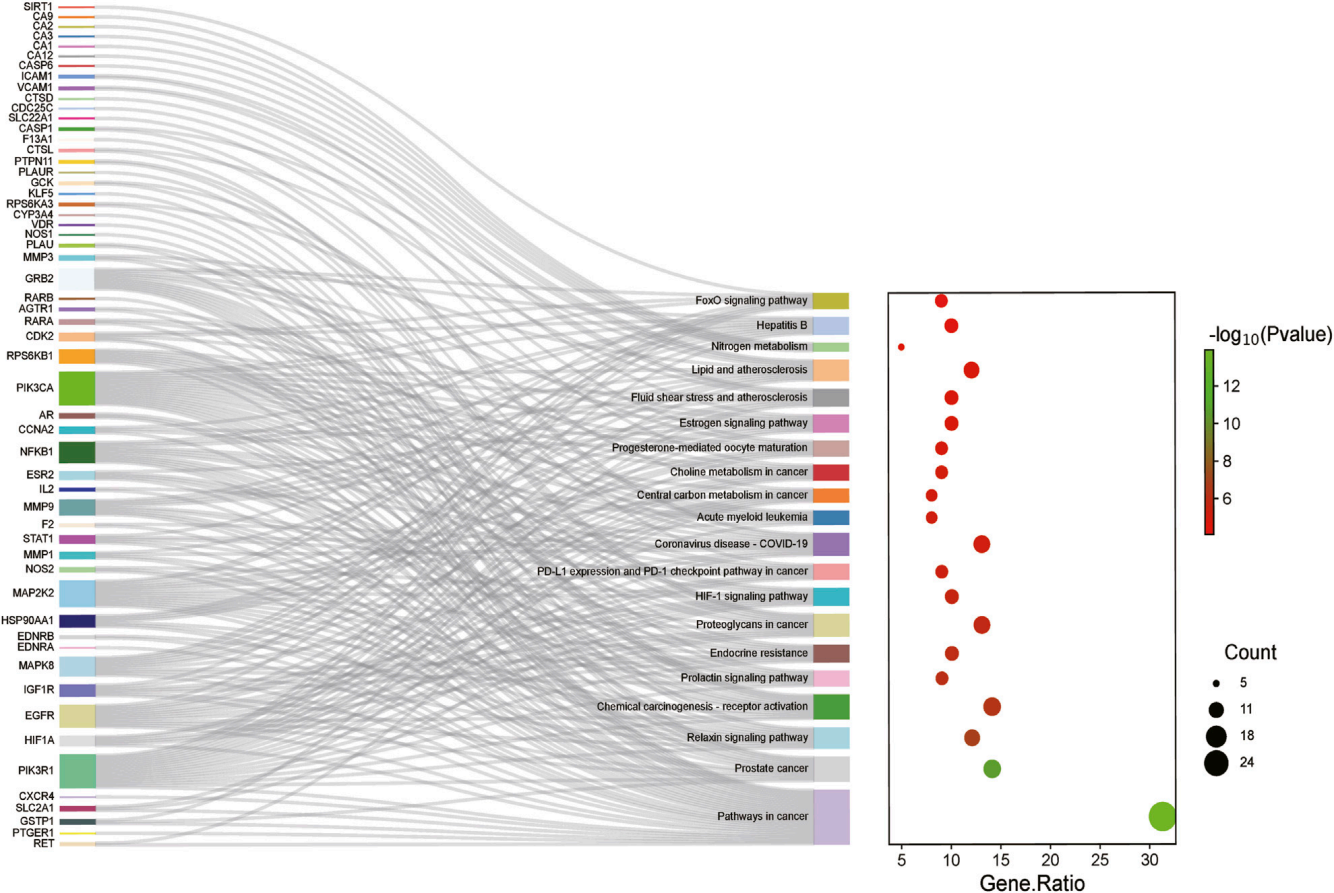
KEGG enrichment analysis of targeted genes of metformin for IM treatment. The color represents the *p*-value, and the spot size represents the number of genes.

The KEGG enrichment analysis indicated that the metformin in IM treatment primarily influenced 80 signaling pathways. The top 20 pathways were charted in Figure 5. Common targeted genes were mostly involved in pathways in tumorigenesis, HIF-1 signaling pathway, PD-L1 expression and PD-1 checkpoint pathway in cancer, FoxO signaling pathway, prolactin signaling pathway, proteoglycans in cancer, and relaxin signaling pathway.

### 3.6 Molecular docking verification

**FIGURE 6:**
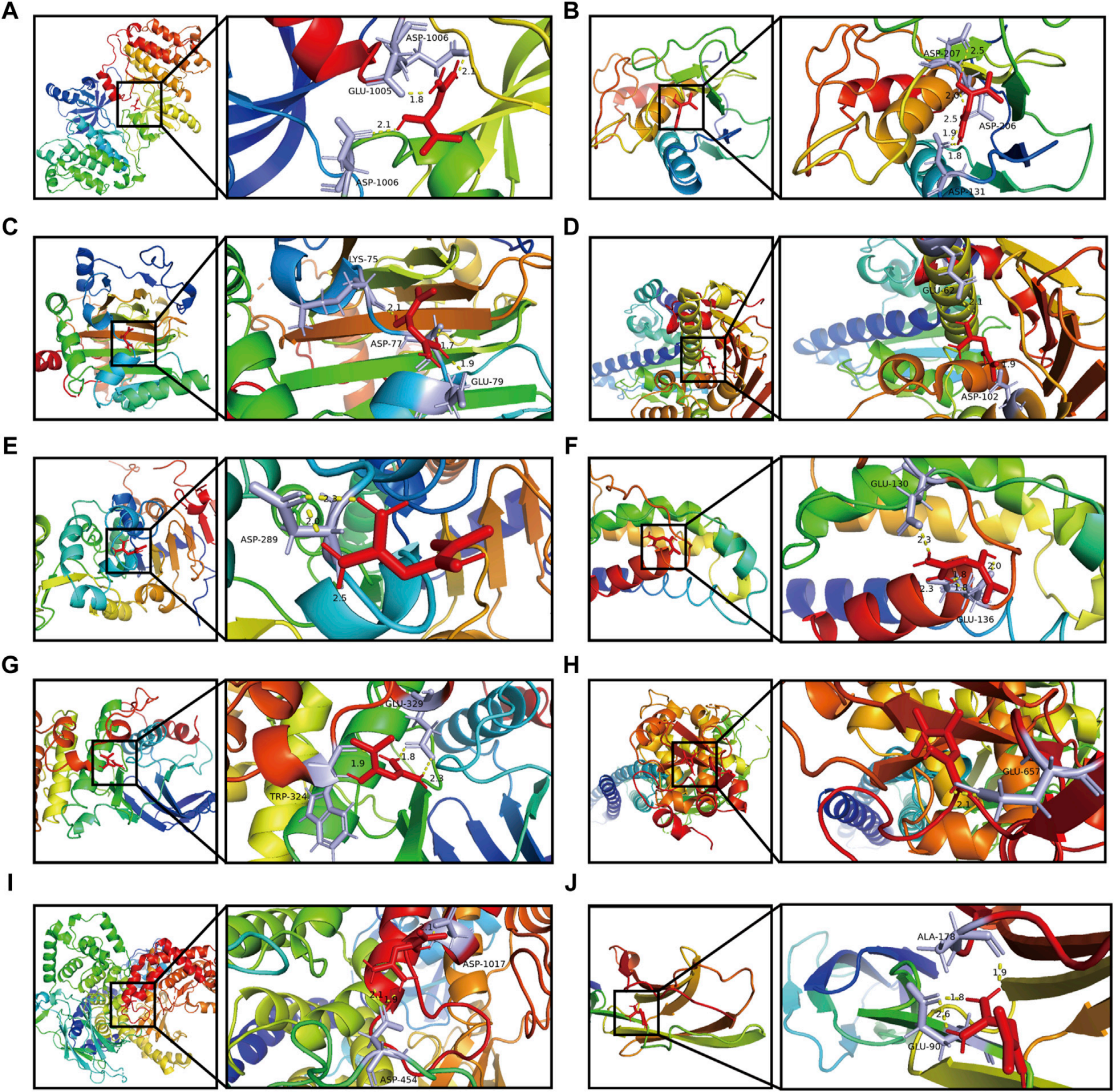
Docking patterns of metformin and its key target proteins. **(A)** Metformin- EGFR. **(B)** Metformin- MMP9. **(C)** Metformin- HIF1A. **(D)** Metformin- HSP90AA1. **(E)** Metformin- SIRT1. **(F)** Metformin- IL2. **(G)** Metformin- MAPK8. **(H)** Metformin- STAT1. **(I)** Metformin- PIK3CA. **(J)** Metformin- ICAM1.

We employed a docking approach to analyse ten hub targets, including EGFR, MMP9, HIF1A, HSP90AA1, SIRT1, IL2, MAPK8, STAT1, PIK3CA, and ICAM1 (Figure 6; Supplementary Figure S2). The information of core target proteins was shown in Supplementary Table S3. The molecular docking analysis revealed that the binding energies between metformin and the target molecules were all below zero (Table 1). Notably, the docking energies between EGFR, MMP9, HIF1A, IL2, PI3KCA and metformin were all less than −5 kcal/Mol.

**TABLE 1:** Summary of metformin and target proteins’ molecular docking.

Molecular name	Targets	Residues	Hydrogen bond length	Binding energy (kcal/Mol)
Metformin	EGFR	ASP-1006; GLU-1005	2.1; 1.8; 2.1	−5.95
Metformin	MMP9	ASP-131; ASP-206; ASP-207	1.8; 1.9; 2.5; 2.0; 2.5	−5.91
Metformin	HIF1A	LYS-75; ASP-77; GLU-79	2.1; 1.7; 1.9	−5.71
Metformin	HSP90AA1	GLU-62; ASP-102	2.1; 1.9	−4.67
Metformin	SIRT1	ASP-289	2.5; 2.3; 2.0	−2.53
Metformin	IL2	GLU-136; GLU-130	1.8; 1.8; 2.0; 2.3; 2.3	−6.18
Metformin	MAPK8	GLU-329; TRP-324	1.8; 2.3; 1.9	−4.82
Metformin	STAT1	GLU-657	2.1	−3.57
Metformin	PIK3CA	ASP-1017; ASP-454	2.1; 2.1; 1.9	−5.51
Metformin	ICAM1	GLU-90; ALA-178	1.8; 2.6; 1.9	−4.45
ANP	EGFR	GLN-791; MET-793; CYS-797; ALA-722; LYS-745; ASP-837; ASN-842; ARG-841	3.0; 2.2; 2.6; 2.0; 2.4; 2.8; 2.3; 2.7; 2.0	−4.54
PZE	MMP9	—	—	−2.71
SO4	HIF1A	—	—	−2.13
AB4	HSP90AA1	ASP-93; THR-104; LYS-58	2.9; 3.0; 2.5	−7.53
GOL	SIRT1	ILE-316; ILE-347	2.2; 1.8	−0.87
SO4	IL2	LYS-74	2.2	−4.14
EDO	MAPK8	GLU-272; ASP-277	3.0; 2.0	−2.29
SO4	STAT1	SER-606; LYS-587; ARG-602	2.9; 2.6; 2.3; 2.1	−5.41
799	PIK3CA	SER-854; GLN-859; VAL-851	2.8; 2.8; 2.8; 1.9	−8.93
NAG	ICAM1	ARG-13; TYR-83; THR-85	2.1; 2.1; 3.1; 3.4	−3.71

Metformin showed a strong association with amino acid residues via the formation of hydrogen bonds. Furthermore, the binding affinities of metformin to EGFR, MMP9, HIF1A, SIRT1, IL2, MAPK8, and ICAM1 were found to be lower than those of the positive control. This observation suggested that metformin performed a strong binding capacity to these target proteins compared. The binding affinities of metformin with STAT1, HSP90AA1, and PIK3CA showed no significant difference from those of the positive control, indicating that metformin’s capacity to bind with these target proteins was nearly the same as that of the positive control.

### 3.7 Metformin inhibited the progression of IM

**FIGURE 7:**
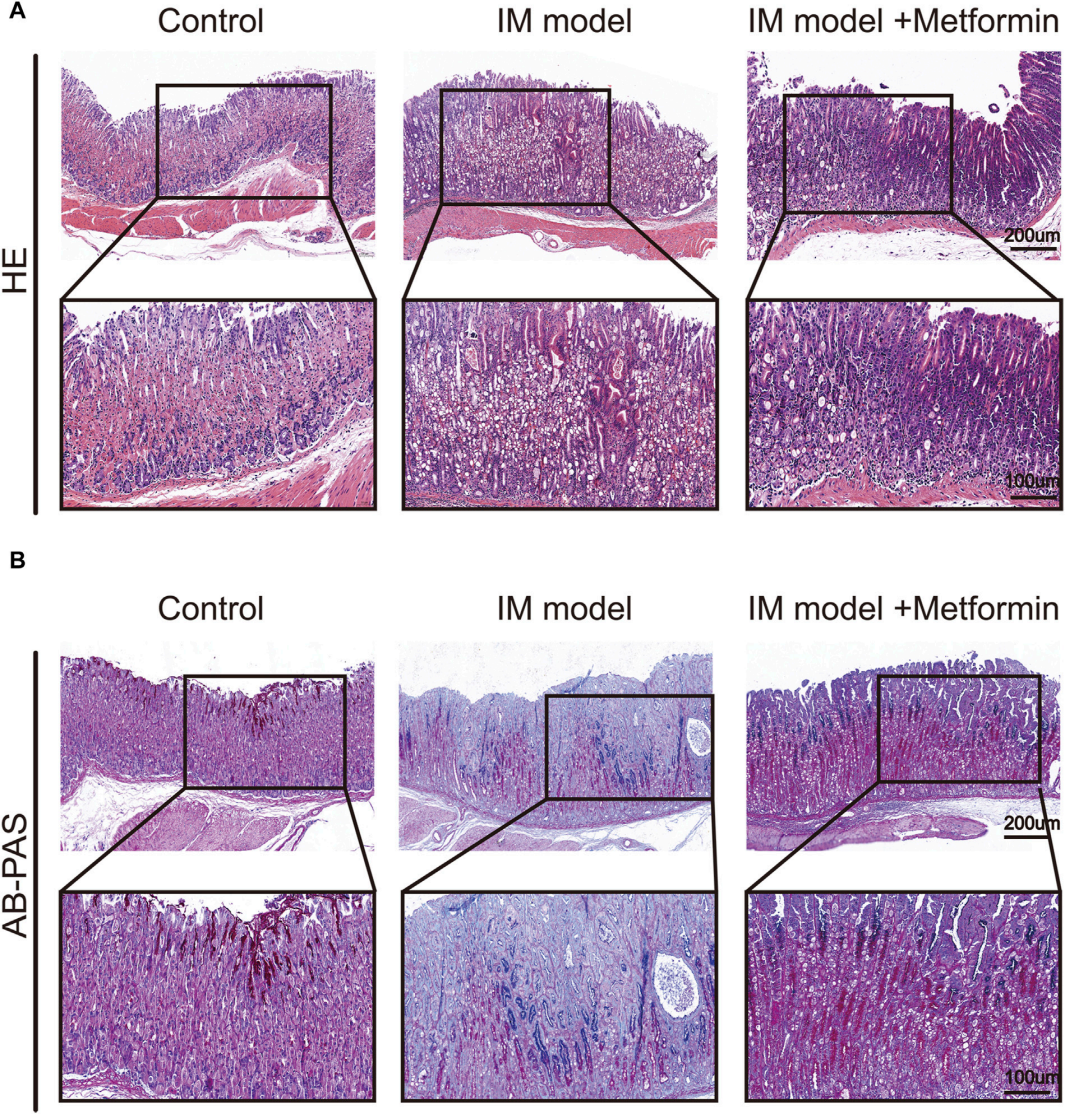
Histopathological changes of gastric mucosa in mice. **(A)** H&E staining (n = 3). **(B)** AB-PAS staining (n = 3).

To examine the impact of metformin, histological slices of gastric tissues were prepared and stained with H&E once the Atp4a^−/−^ mice reached the age of 24 weeks. In the control group, the gastric mucosal epithelial cells were neatly distributed, the glandular structure was normal and tightly arranged, and no pathological changes were observed. Atp4a^−/−^ mice showed disordered distribution of gastric mucosal epithelial cells, loss of parietal cells, the appearance of goblet cells, inflammatory cell infiltration, and disordered glandular structures. In the metformin-treated group, the inflammatory cell infiltration was reduced, glandular structures tended to be regular, and the pathological changes were less severe than those in the Atp4a^−/−^ model group (Figure 7A). The level of IM lesions in gastric tissues was assessed using AB-PAS staining, a technique that involves the staining of neutral mucins in red and sialomucins in blue. The presence of IM lesions in the gastric mucosa of Atp4a^−/−^ mice was observed to be extensive compared with normal mucosa. In metformin-treated Atp4a^−/−^ mice, IM lesions were regressed apparently (Figure 7B), suggesting that metformin reduced the lesion of gastric mucosa.

### 3.8 Metformin regulates the expression pattern of IM markers

**FIGURE 8:**
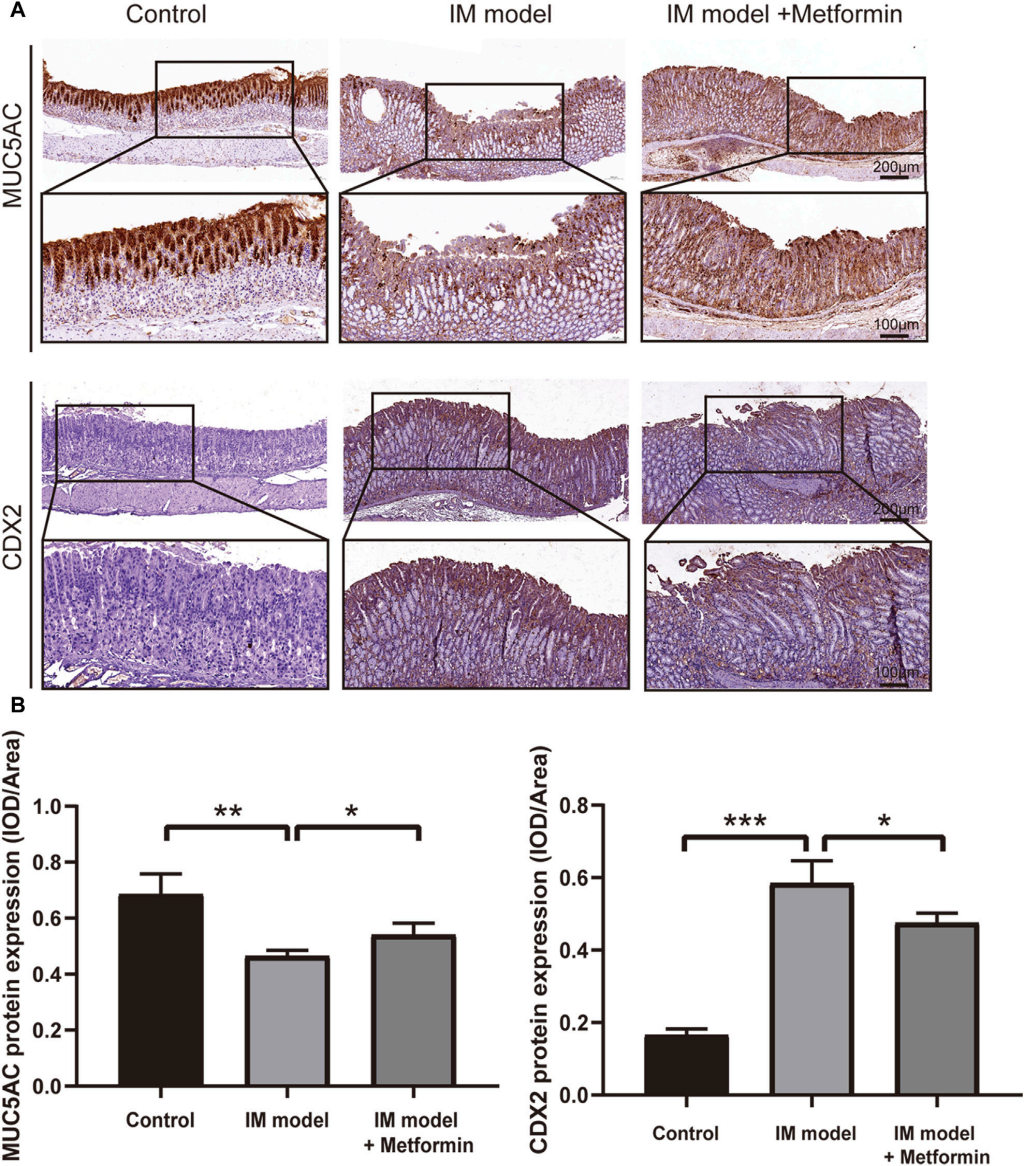
Metformin increases the level of MUC5AC and inhibits the expression of CDX2 in Atp4a^−/−^ mice. **(A)** The expression of MUC5AC and CDX2 in the Atp4a^−/−^ mice gastric tissues using IHC staining (n = 3). **(B)** The quantification of relative protein levels was performed using optical density analysis with ImageJ software. (*p < 0.05, **p < 0.01, ****p < 0.0001).

To further understand the characters of IM tissues in Atp4a^−/−^ mice and investigate the effect of metformin on IM, IHC staining was used to determine the expression of MUC5AC and CDX2 (Figure 8A). In the control group, its gastric mucosal epithelium showed strong positive expression of MUC5AC, with deep staining and wide distribution in the whole epithelium. The expression of MUC5AC in gastric mucosal epithelium of Atp4a^−/−^ mice was significantly lower than that of the control group. After metformin treatment, the positive expression of MUC5AC increased significantly compared with the model group. The negative expression of CDX2 was observed in gastric mucosa in the control group. The expression of CDX2 in gastric mucosa of Atp4a^−/−^ model mice was significantly increased compared with that of controls. Positive expression of CDX2 protein was significantly reduced in the metformin-treated group (Figure 8B). Thus, the metformin treatment can effectively increase the expression of MUC5AC and reduce the expression of CDX2.

### 3.9 Metformin treatment regulated the core- and related-targets

**FIGURE 9:**
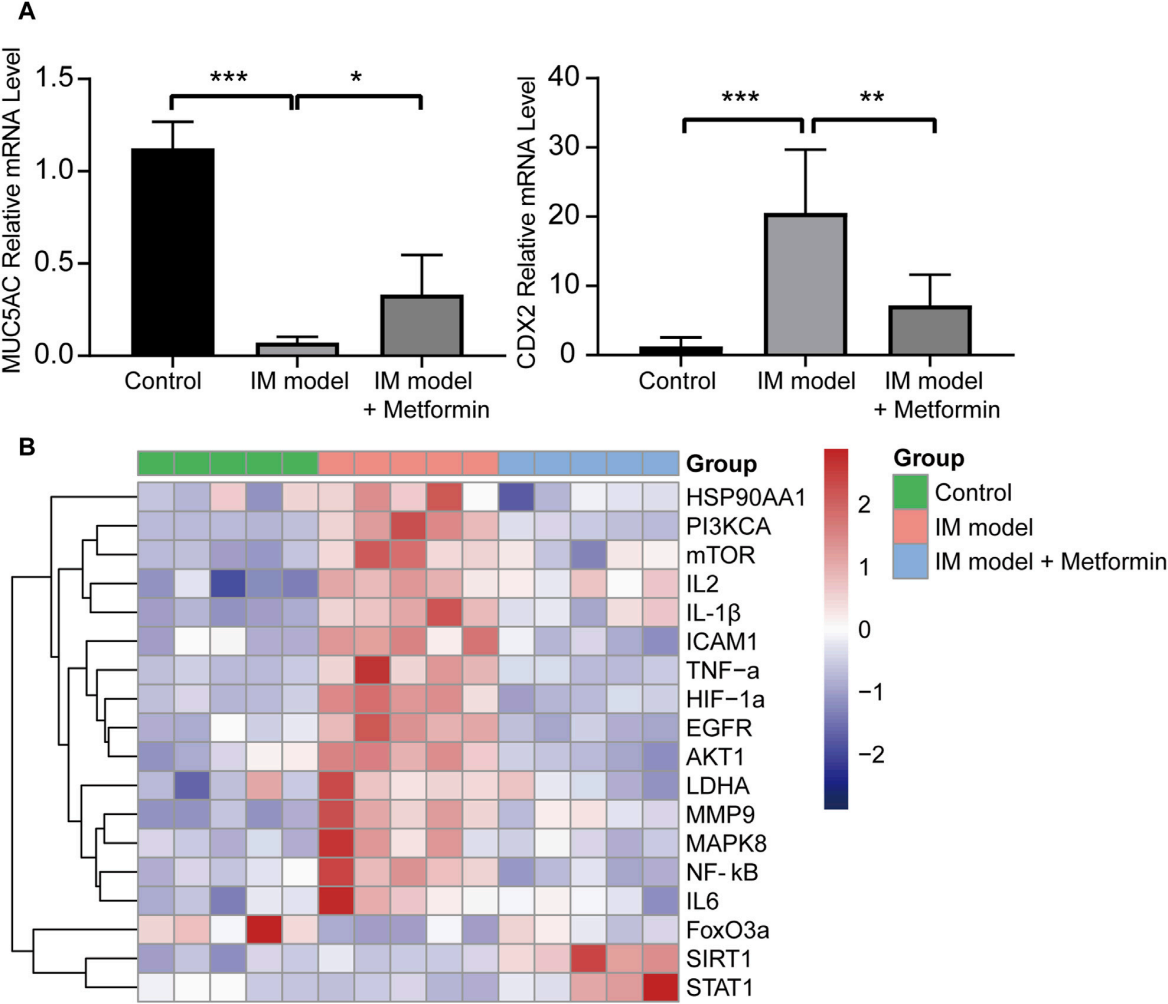
The mRNA expression of core targets and related factors. **(A)** The relative mRNA expression levels of MUC5AC and CDX2 (n = 5). **(B)** The heatmap of the relative mRNA expression levels of core target genes and pathway-related genes in three groups (n = 5). (*p < 0.05, **p < 0.01, ***p < 0.001, ****p < 0.0001).

The qRT-PCR results indicated that metformin increased the expression of *MUC5AC* and decreased the expression of *CDX2* significantly (Figure 9A), which further verified the IHC staining results in Figure 8B. It was further proved that metformin could effectively inhibit the degree of intestinal metaplasia in the gastric mucosa of Atp4a^−/−^ mice. Compared with the wide type mice, the mRNA expression of *EGFR*, *MMP9*, *HIF1A*, *HSP90AA1*, *MAPK8*, *ICAM1*, *IL2*, *NF-κB*, *IL6*, *IL-1β*, *TNF-α*, *PI3KCA*, *AKT1*, *mTOR*, and *LDHA* increased significantly, while the mRNA expression of *STAT1* and *FoxO3a* decreased significantly in the gastric tissues of Atp4a^−/−^ mice. Furthermore, the treatment of metformin in Atp4a^−/−^ mice drive the expressions of these targets the other way around significantly. The expression of *SIRT1* was increased significantly following the metformin intervention, although the difference between the normal and model groups was not significant (Figure 9B).

### 3.10 The effect of metformin in NF-κB and PI3K/AKT/mTOR/HIF-1α signaling pathway

**FIGURE 10:**
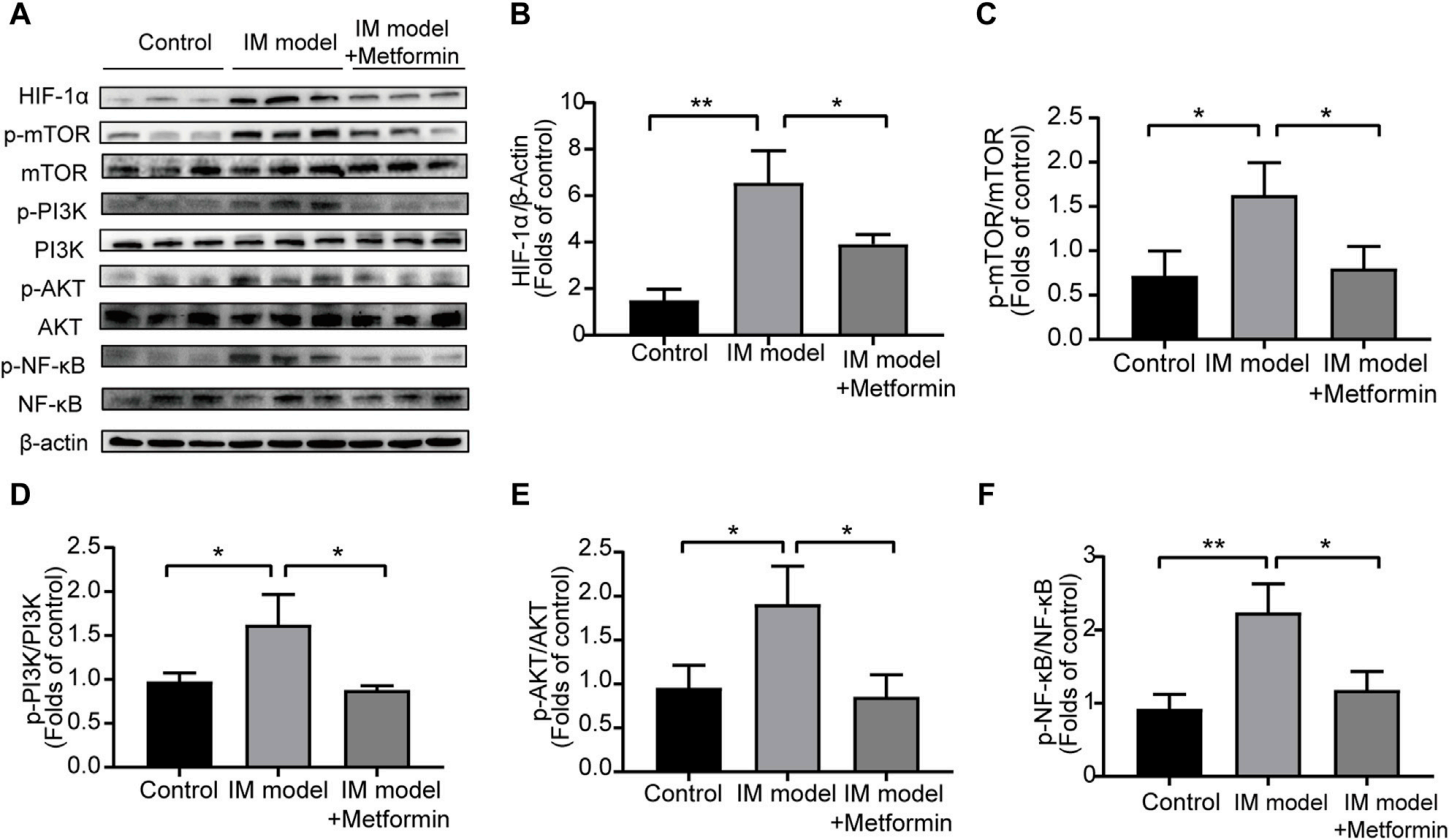
Metformin reduced the activation of NF-κB and inhibited the PI3K/AKT/mTOR/HIF-1α signaling pathway in the gastric tissues of Atp4a^−/−^ mice. **(A)** Western blot was conducted to assess the protein levels of NF-κB, phospho-NF-κB, PI3K, phospho-PI3K, AKT, phospho-AKT, mTOR, phospho-mTOR, and HIF-1α (n = 3). **(B–F)** The relative protein levels were quantified (n = 3). (*p < 0.05, **p < 0.01).

The Western blot analysis was conducted to evaluate the protein levels of NF-κB and PI3K/AKT/mTOR/HIF-1α signaling pathway in gastric tissues of Atp4a^−/−^ mice (Figure 10A). Compared with the control group, the expression of p-NF-κB, p-PI3K, p-Akt, p-mTOR, and HIF-1α in IM mice increased significantly, indicating the activation of NF-κB and the PI3K/AKT/mTOR/HIF-1α signaling pathway in the progression of IM. The results of metformin-treated group demonstrated that metformin inhibited the expression of p-NF-κB, p-PI3K, p-AKT, p-mTOR, and HIF-1α significantly (Figures 10B–E). These findings suggested that metformin could lower the activation of NF-κB and inhibit the PI3K/AKT/mTOR/HIF-1α signaling pathway in Atp4a^−/−^ mice.

## 4 Discussion

IM-related genes were mainly concentrated in interleukins related pathways and apoptosis, while targeted genes of metformin were involved in transduction pathways and interleukins signaling pathways. Using network pharmacological analysis, 1,751 IM-related genes and 318 metformin-targeted genes were identified, and 99 common genes were identified. Common genes were mainly involved in the pathways in cancer, HIF-1 signaling pathway, PD-L1 expression and PD-1 checkpoint pathway in cancer, FoxO signaling pathway. Ten core targets (EGFR, MMP9, HIF1A, HSP90AA1, SIRT1, IL2, MAPK8, STAT1, PIK3CA and ICAM1) identified in PPI analysis exhibited a strong correlation with the therapeutic effects of metformin in IM treatment. The molecular docking results demonstrated that metformin exhibited spontaneous binding to core target proteins, indicating its potential role in modulating the biological activity of these core targets. Besides, metformin treatment regulated the expressions of core targets and related factors in the IM mice model.

Metformin can increase the radiosensitivity of cancer cells by down-regulating EGFR/PI3K/AKT (Zhang et al., 2014; Song et al., 2015). The PI3K/AKT/mTOR signaling pathway is known to facilitate the proliferation of cancer cells, and it has been observed that metformin possesses the ability to impede the activity of this pathway (Ala and Ala, 2021). The activation of the PI3K/AKT pathway has been found to induce the activation of NF-κB and the inactivation of FoxO3a, eventually facilitating the advancement of gastric cancer (Huang et al., 2017; Rena et al., 2017). FoxO3a can directly increase its transcriptional activity by phosphorylating AMPK (Greer et al., 2007). Furthermore, the decrease in FoxO3a expression results in a notable elevation in NF-κB activity and the production of inflammatory factors (Lin et al., 2004). In parallel, metformin improves the immune response to cancer cells by reducing NF-κB (Kheirandish et al., 2018). It inhibits the phosphorylation and nuclear translocation of the NF-κB subunit p65 and suppresses the degradation of its inhibitory protein I καB. This process leads to NF-κB being sequestered in the cytoplasm and unable to translocate to the nucleus to participate in inducing an inflammatory response, which reduces the upregulation of IL-1, IL-6, IL-2 and TNF-α (Feng et al., 2023). Metformin has been found to possess the capability to diminish the production of HIF-1α via AMPK activation, leading to a decrease in tumor metastasis (Meireles et al., 2022). Metformin can enhance the AMPK/mTOR pathway-dependent expression of STAT1, thus promoting the immunoregulatory effect. In intestinal inflammation, the drug also negatively regulates heat shock protein 90 (HSP90) (Saber and El-Kader, 2021). The previous study showed that metformin can exert inhibitory effects on cellular migration and invasion by regulating MMP9 activity (Jang et al., 2014; Cheng et al., 2022). Metformin also reduces the expression of ICAM1, thus inhibiting inflammatory signaling and metastatic progression (Liu et al., 2014; Schexnayder et al., 2018).

Loss of H + -K + -ATPase α subunit leads to parietal cell loss resulting in loss of gastric acid, increased number of glands, and vesicle formation in Atp4a^−/−^ mice at 12 weeks of age, while chronic inflammation leads to atrophy of the basal mucosa and development of IM (Judd et al., 2005; Liu et al., 2020). In the present study, we selected Atp4a^−/−^ mice as the animal model and revealed metformin may have a therapeutic effect on IM *in vivo*, which provides firm evidence for our prediction and analysis. Studies have confirmed that insufficient expression of MUC5AC and ectopic expression of CDX2 could be observed in IM (Battista et al., 2021; Koide et al., 2022). The IHC staining results of MUC5AC and CDX2 provided further validation of the therapeutic effect of metformin in IM. After metformin treatment, the expressions of inflammatory factors such as IL-1β, TNF-α, IL-6 and IL-2 were reduced and the activation of NF-κB was decreased, suggesting that metformin reduced the inflammation in Atp4a^−/−^ mice. Additionally, Western blot results demonstrated that metformin can stop the further progression of IM by inhibiting PI3K/Akt/mTOR/HIF-1α signaling pathway. Combined with the enrichment analysis results, animal experiments indicated that metformin may participate in apoptosis and inflammation to slow down the IM process.

Although Atp4a^−/−^ mice can mimic the pathological changes and molecular characteristics of IM, in extreme cases, H + -K + -ATPase α subunit deletion can also cause dysfunction in other organs, which is different from human gastric IM. While molecular docking is a valuable tool, other additional validation methods are needed to further confirm the interactions between metformin and its targets. The downstream factors of the selected signaling pathway need to be further explored. In parallel, this study provides an explanation and theoretical basis for the potential chemopreventive effect of metformin on gastric cancer in clinical trials. Clinical trials of metformin in human gastric IM patients should be performed and investigated to further validate the experimental results and lay a foundation for the clinical application of metformin.

## 5 Conclusion

This study demonstrates that metformin has a therapeutic effect on IM in Atp4a^−/−^ mice. Combining the results of network pharmacology and molecular docking, it regulates the expressions of predicted core targets and related factors. Besides, metformin reduces inflammation and inhibits the PI3K/AKT/mTOR/HIF-1α signaling pathway, which eventually slows down the IM process in inflammation and apoptosis.

## Data Availability

The original contributions presented in the study are included in the article/Supplementary Material, further inquiries can be directed to the corresponding authors.

## Supplementary material

SUPPLEMENTARY FIGURE S1 GO enrichment analyses of IM-related genes and metformin-targeted genes. **(A)** Top ten cellular components of IM-related genes. **(B)** Top ten molecular functions of IM-related genes. **(C)** Top ten biological processes of IM-related genes. **(D)** Top ten cellular components of metformin-targeted genes. **(E)** Top ten molecular functions of metformin-targeted genes. **(F)** Top ten biological processes of metformin-targeted gene.

SUPPLEMENTARY FIGURE S2 Patterns of molecular docking between the primary target proteins and the original ligands. **(A)** ANP- EGFR. **(B)** PZE-MMP9. **(C)** SO4- HIF1A. **(D)** AB4- HSP90AA1. **(E)** GOL- SIRT1. **(F)** SO4- IL2. **(G)** EDO- MAPK8. **(H)** SO4- STAT1. **(I)** 799- PIK3CA. **(J)** NAG-ICAM1.

SUPPLEMENTARY TABLE S1 The databases information and their websites.

SUPPLEMENTARY TABLE S2 The primer sequences of targets genes and related genes.

SUPPLEMENTARY TABLE S3 The core targets proteins information.

## References

[B1] Ala M. Ala M. (2021). Metformin for cardiovascular protection, inflammatory bowel disease, osteoporosis, periodontitis, polycystic ovarian syndrome, neurodegeneration, cancer, inflammation and senescence: what is next? ACS Pharmacol. Transl. Sci. 4, 1747–1770. 10.1021/acsptsci.1c00167 34927008 PMC8669709

[B2] Battista S. Ambrosio M. R. Limarzi F. Gallo G. Saragoni L. (2021). Molecular alterations in gastric preneoplastic lesions and early gastric cancer. Int. J. Mol. Sci. 22, 6652. 10.3390/ijms22136652 34206291 PMC8268370

[B3] Ba X. Huang Y. Shen P. Huang Y. Wang H. Han L. (2021). WTD attenuating rheumatoid arthritis *via* suppressing angiogenesis and modulating the PI3K/AKT/mTOR/HIF-1α pathway. Front. Pharmacol. 12, 696802. 10.3389/fphar.2021.696802 34646130 PMC8502817

[B4] Berkman S. J. Roscoe E. M. Bourret J. C. (2019). Comparing self-directed methods for training staff to create graphs using Graphpad Prism. J. Appl. Behav. Anal. 52, 188–204. 10.1002/jaba.522 30382580

[B5] Cheng J. Li C. Ying Y. Lv J. Qu X. Mcgowan E. (2022). Metformin alleviates endometriosis and potentiates endometrial receptivity via decreasing VEGF and MMP9 and increasing leukemia inhibitor factor and HOXA10. Front. Pharmacol. 13, 750208. 10.3389/fphar.2022.750208 35273494 PMC8902464

[B6] Cheung K. S. Chan E. W. Wong A. Y. S. Chen L. Seto W. K. Wong I. C. K. (2019). Metformin use and gastric cancer risk in diabetic patients after *Helicobacter pylori* eradication. J. Natl. Cancer Inst. 111, 484–489. 10.1093/jnci/djy144 30329127

[B7] Cheung K. S. Chung K. L. Leung W. K. (2022). Chemopreventive effect of metformin on gastric cancer development. Gut Liver 16, 147–156. 10.5009/gnl210132 34158423 PMC8924804

[B8] Choi S. I. Yoon C. Park M. R. Lee D. Kook M. C. Lin J. X. (2019). CDX1 expression induced by CagA-expressing *Helicobacter pylori* promotes gastric tumorigenesis. Mol. Cancer Res. 17, 2169–2183. 10.1158/1541-7786.MCR-19-0181 31416838

[B9] Choi E. Hendley A. M. Bailey J. M. Leach S. D. Goldenring J. R. (2016). Expression of activated Ras in gastric chief cells of mice leads to the full spectrum of metaplastic lineage transitions. Gastroenterology 150, 918–930. 10.1053/j.gastro.2015.11.049 26677984 PMC4808451

[B10] Correa P. Piazuelo M. B. (2012). The gastric precancerous cascade. J. Dig. Dis. 13, 2–9. 10.1111/j.1751-2980.2011.00550.x 22188910 PMC3404600

[B11] Feng Y. Y. Wang Z. Pang H. (2023). Role of metformin in inflammation. Mol. Biol. Rep. 50, 789–798. 10.1007/s11033-022-07954-5 36319785

[B12] Giroux V. Rustgi A. K. (2017). Metaplasia: tissue injury adaptation and a precursor to the dysplasia-cancer sequence. Nat. Rev. Cancer 17, 594–604. 10.1038/nrc.2017.68 28860646 PMC5998678

[B13] Greer E. L. Oskoui P. R. Banko M. R. Maniar J. M. Gygi M. P. Gygi S. P. (2007). The energy sensor AMP-activated protein kinase directly regulates the mammalian FOXO3 transcription factor. J. Biol. Chem. 282, 30107–30119. 10.1074/jbc.M705325200 17711846

[B14] Hayakawa Y. Ariyama H. Stancikova J. Sakitani K. Asfaha S. Renz B. W. (2015). Mist1 expressing gastric stem cells maintain the normal and neoplastic gastric epithelium and are supported by a perivascular stem cell niche. Cancer Cell. 28, 800–814. 10.1016/j.ccell.2015.10.003 26585400 PMC4684751

[B15] Hayakawa Y. Nakagawa H. Rustgi A. K. Que J. Wang T. C. (2021). Stem cells and origins of cancer in the upper gastrointestinal tract. Cell. Stem Cell. 28, 1343–1361. 10.1016/j.stem.2021.05.012 34129814 PMC8844710

[B16] He S. Wang T. Shi C. Wang Z. Fu X. (2022). Network pharmacology-based approach to understand the effect and mechanism of Danshen against anemia. J. Ethnopharmacol. 282, 114615. 10.1016/j.jep.2021.114615 34509606

[B17] Huang Y. Zhang J. Hou L. Wang G. Liu H. Zhang R. (2017). LncRNA AK023391 promotes tumorigenesis and invasion of gastric cancer through activation of the PI3K/Akt signaling pathway. J. Exp. Clin. Cancer Res. 36, 194. 10.1186/s13046-017-0666-2 29282102 PMC5745957

[B18] Jang S. Y. Kim A. Kim J. K. Kim C. Cho Y. H. Kim J. H. (2014). Metformin inhibits tumor cell migration via down-regulation of MMP9 in tamoxifen-resistant breast cancer cells. Anticancer Res. 34, 4127–4134. 25075039

[B19] Jiao W. Mi S. Sang Y. Jin Q. Chitrakar B. Wang X. (2022). Integrated network pharmacology and cellular assay for the investigation of an anti-obesity effect of 6-shogaol. Food Chem. 374, 131755. 10.1016/j.foodchem.2021.131755 34883426

[B20] Judd L. M. Andringa A. Rubio C. A. Spicer Z. Shull G. E. Miller M. L. (2005). Gastric achlorhydria in H/K-ATPase-deficient (Atp4a(-/-)) mice causes severe hyperplasia, mucocystic metaplasia and upregulation of growth factors. J. Gastroenterol. Hepatol. 20, 1266–1278. 10.1111/j.1440-1746.2005.03867.x 16048577

[B21] Kheirandish M. Mahboobi H. Yazdanparast M. Kamal W. Kamal M. A. (2018). Anti-cancer effects of metformin: recent evidences for its role in prevention and treatment of cancer. Curr. Drug Metab. 19, 793–797. 10.2174/1389200219666180416161846 29663879

[B22] Koide T. Koyanagi-Aoi M. Uehara K. Kakeji Y. Aoi T. (2022). CDX2-induced intestinal metaplasia in human gastric organoids derived from induced pluripotent stem cells. iScience 25, 104314. 10.1016/j.isci.2022.104314 35602937 PMC9118752

[B23] Lamoia T. E. Shulman G. I. (2021). Cellular and molecular mechanisms of metformin action. Endocr. Rev. 42, 77–96. 10.1210/endrev/bnaa023 32897388 PMC7846086

[B24] Lin L. Hron J. D. Peng S. L. (2004). Regulation of NF-kappaB, Th activation, and autoinflammation by the forkhead transcription factor Foxo3a. Immunity 21, 203–213. 10.1016/j.immuni.2004.06.016 15308101

[B25] Liu W. Yang L. J. Liu Y. L. Yuan D. S. Zhao Z. M. Wang Q. (2020). Dynamic characterization of intestinal metaplasia in the gastric corpus mucosa of Atp4a-deficient mice. Biosci. Rep. 40. 10.1042/BSR20181881 PMC7040465 31904088

[B26] Liu Y. Tang G. Li Y. Wang Y. Chen X. Gu X. (2014). Metformin attenuates blood-brain barrier disruption in mice following middle cerebral artery occlusion. J. Neuroinflammation 11, 177. 10.1186/s12974-014-0177-4 25315906 PMC4201919

[B27] Li X. Wei S. Niu S. Ma X. Li H. Jing M. (2022). Network pharmacology prediction and molecular docking-based strategy to explore the potential mechanism of Huanglian Jiedu Decoction against sepsis. Comput. Biol. Med. 144, 105389. 10.1016/j.compbiomed.2022.105389 35303581

[B28] Li Y. Yang L. Wang Y. Deng Z. Xu S. Xie H. (2021). Exploring metformin as a candidate drug for rosacea through network pharmacology and experimental validation. Pharmacol. Res. 174, 105971. 10.1016/j.phrs.2021.105971 34763093

[B29] Martin-Montalvo A. Mercken E. M. Mitchell S. J. Palacios H. H. Mote P. L. Scheibye-Knudsen M. (2013). Metformin improves healthspan and lifespan in mice. Nat. Commun. 4, 2192. 10.1038/ncomms3192 23900241 PMC3736576

[B30] Ma X. Zhao Y. Yang T. Gong N. Chen X. Liu G. (2022). Integration of network pharmacology and molecular docking to explore the molecular mechanism of Cordycepin in the treatment of Alzheimer's disease. Front. Aging Neurosci. 14, 1058780. 10.3389/fnagi.2022.1058780 36620771 PMC9817107

[B31] Meireles C. G. Lourenco de Lima C. Martins de Paula Oliveira M. Abe Da Rocha Miranda R. Romano L. Yo-Stella Brashaw T. (2022). Antiproliferative effects of metformin in cellular models of pheochromocytoma. Mol. Cell. Endocrinol. 539, 111484. 10.1016/j.mce.2021.111484 34637881

[B32] Miao Z. F. Adkins-Threats M. Burclaff J. R. Osaki L. H. Sun J. X. Kefalov Y. (2020). A metformin-responsive metabolic pathway controls distinct steps in gastric progenitor fate decisions and maturation. Cell. Stem Cell. 26, 910–925. 10.1016/j.stem.2020.03.006 32243780 PMC7275895

[B33] Nag A. Dhull N. Gupta A. (2023). Evaluation of tea (Camellia sinensis L.) phytochemicals as multi-disease modulators, a multidimensional *in silico* strategy with the combinations of network pharmacology, pharmacophore analysis, statistics and molecular docking. Mol. Divers 27, 487–509. 10.1007/s11030-022-10437-1 35536529 PMC9086669

[B34] Nam K. T. Lee H. J. Sousa J. F. Weis V. G. O'Neal R. L. Finke P. E. (2010). Mature chief cells are cryptic progenitors for metaplasia in the stomach. Gastroenterology 139, 2028–2037. 10.1053/j.gastro.2010.09.005 20854822 PMC2997152

[B35] Rena G. Hardie D. G. Pearson E. R. (2017). The mechanisms of action of metformin. Diabetologia 60, 1577–1585. 10.1007/s00125-017-4342-z 28776086 PMC5552828

[B36] Saber S. El-Kader E. M. A. (2021). Novel complementary coloprotective effects of metformin and MCC950 by modulating HSP90/NLRP3 interaction and inducing autophagy in rats. Inflammopharmacology 29, 237–251. 10.1007/s10787-020-00730-6 32594364

[B37] Schexnayder C. Broussard K. Onuaguluchi D. Poche A. Ismail M. Mcatee L. (2018). Metformin inhibits migration and invasion by suppressing ROS production and COX2 expression in MDA-MB-231 breast cancer cells. Int. J. Mol. Sci. 19, 3692. 10.3390/ijms19113692 30469399 PMC6274682

[B38] Schindelin J. Arganda-Carreras I. Frise E. Kaynig V. Longair M. Pietzsch T. (2012). Fiji: an open-source platform for biological-image analysis. Nat. Methods 9, 676–682. 10.1038/nmeth.2019 22743772 PMC3855844

[B39] Shang L. Wang Y. Li J. Zhou F. Xiao K. Liu Y. (2023). Mechanism of Sijunzi Decoction in the treatment of colorectal cancer based on network pharmacology and experimental validation. J. Ethnopharmacol. 302, 115876. 10.1016/j.jep.2022.115876 36343798

[B40] Song Y. M. Lee Y. H. Kim J. W. Ham D. S. Kang E. S. Cha B. S. (2015). Metformin alleviates hepatosteatosis by restoring SIRT1-mediated autophagy induction via an AMP-activated protein kinase-independent pathway. Autophagy 11, 46–59. 10.4161/15548627.2014.984271 25484077 PMC4502778

[B41] Strong R. Miller R. A. Antebi A. Astle C. M. Bogue M. Denzel M. S. (2016). Longer lifespan in male mice treated with a weakly estrogenic agonist, an antioxidant, an α-glucosidase inhibitor or a Nrf2-inducer. Aging Cell. 15, 872–884. 10.1111/acel.12496 27312235 PMC5013015

[B42] Sung H. Ferlay J. Siegel R. L. Laversanne M. Soerjomataram I. Jemal A. (2021). Global cancer statistics 2020: GLOBOCAN estimates of incidence and mortality worldwide for 36 cancers in 185 countries. CA Cancer J. Clin. 71, 209–249. 10.3322/caac.21660 33538338

[B43] Tatematsu M. Tsukamoto T. Inada K. (2003). Stem cells and gastric cancer: role of gastric and intestinal mixed intestinal metaplasia. Cancer Sci. 94, 135–141. 10.1111/j.1349-7006.2003.tb01409.x 12708487 PMC11160206

[B44] Wang Y. Yuan Y. Wang W. He Y. Zhong H. Zhou X. (2022). Mechanisms underlying the therapeutic effects of Qingfeiyin in treating acute lung injury based on GEO datasets, network pharmacology and molecular docking. Comput. Biol. Med. 145, 105454. 10.1016/j.compbiomed.2022.105454 35367781

[B45] Zhang D. Wang Z. Li J. Zhu J. (2022a). Exploring the possible molecular targeting mechanism of Saussurea involucrata in the treatment of COVID-19 based on bioinformatics and network pharmacology. Comput. Biol. Med. 146, 105549. 10.1016/j.compbiomed.2022.105549 35751193 PMC9035664

[B46] Zhang H. Zhang Y. Li Y. Wang Y. Yan S. Xu S. (2021a). Bioinformatics and network pharmacology identify the therapeutic role and potential mechanism of melatonin in AD and rosacea. Front. Immunol. 12, 756550. 10.3389/fimmu.2021.756550 34899707 PMC8657413

[B47] Zhang J. Fan F. Liu A. Zhang C. Li Q. Zhang C. (2022b). Icariin: a potential molecule for treatment of knee osteoarthritis. Front. Pharmacol. 13, 811808. 10.3389/fphar.2022.811808 35479319 PMC9037156

[B48] Zhang L. Han L. Wang X. Wei Y. Zheng J. Zhao L. (2021b). Exploring the mechanisms underlying the therapeutic effect of Salvia miltiorrhiza in diabetic nephropathy using network pharmacology and molecular docking. Biosci. Rep. 41. 10.1042/BSR20203520 PMC8209169 33634308

[B49] Zhang T. Zhang L. Zhang T. Fan J. Wu K. Guan Z. (2014). Metformin sensitizes prostate cancer cells to radiation through EGFR/p-DNA-PKCS *in vitro* and *in vivo* . Radiat. Res. 181, 641–649. 10.1667/RR13561.1 24844651

[B50] Zhang W. Yang Z. Zhou F. Wei Y. Ma X. (2022c). Network pharmacology and bioinformatics analysis identifies potential therapeutic targets of paxlovid against LUAD/COVID-19. Front. Endocrinol. (Lausanne) 13, 935906. 10.3389/fendo.2022.935906 36157452 PMC9493477

[B51] Zhou W. Zhang H. Wang X. Kang J. Guo W. Zhou L. (2022). Network pharmacology to unveil the mechanism of Moluodan in the treatment of chronic atrophic gastritis. Phytomedicine 95, 153837. 10.1016/j.phymed.2021.153837 34883416

[B52] Zhu W. Li Y. Zhao J. Wang Y. Li Y. Wang Y. (2022). The mechanism of triptolide in the treatment of connective tissue disease-related interstitial lung disease based on network pharmacology and molecular docking. Ann. Med. 54, 541–552. 10.1080/07853890.2022.2034931 35132912 PMC8843192

