# Supplementary figures and images for: Revealing the role of metformin in gastric intestinal metaplasia treatment
Source: Front Pharmacol. 2024 Jul 19;15:1340309. doi: 10.3389/fphar.2024.1340309 (PMC11294171; doi:10.3389/fphar.2024.1340309)

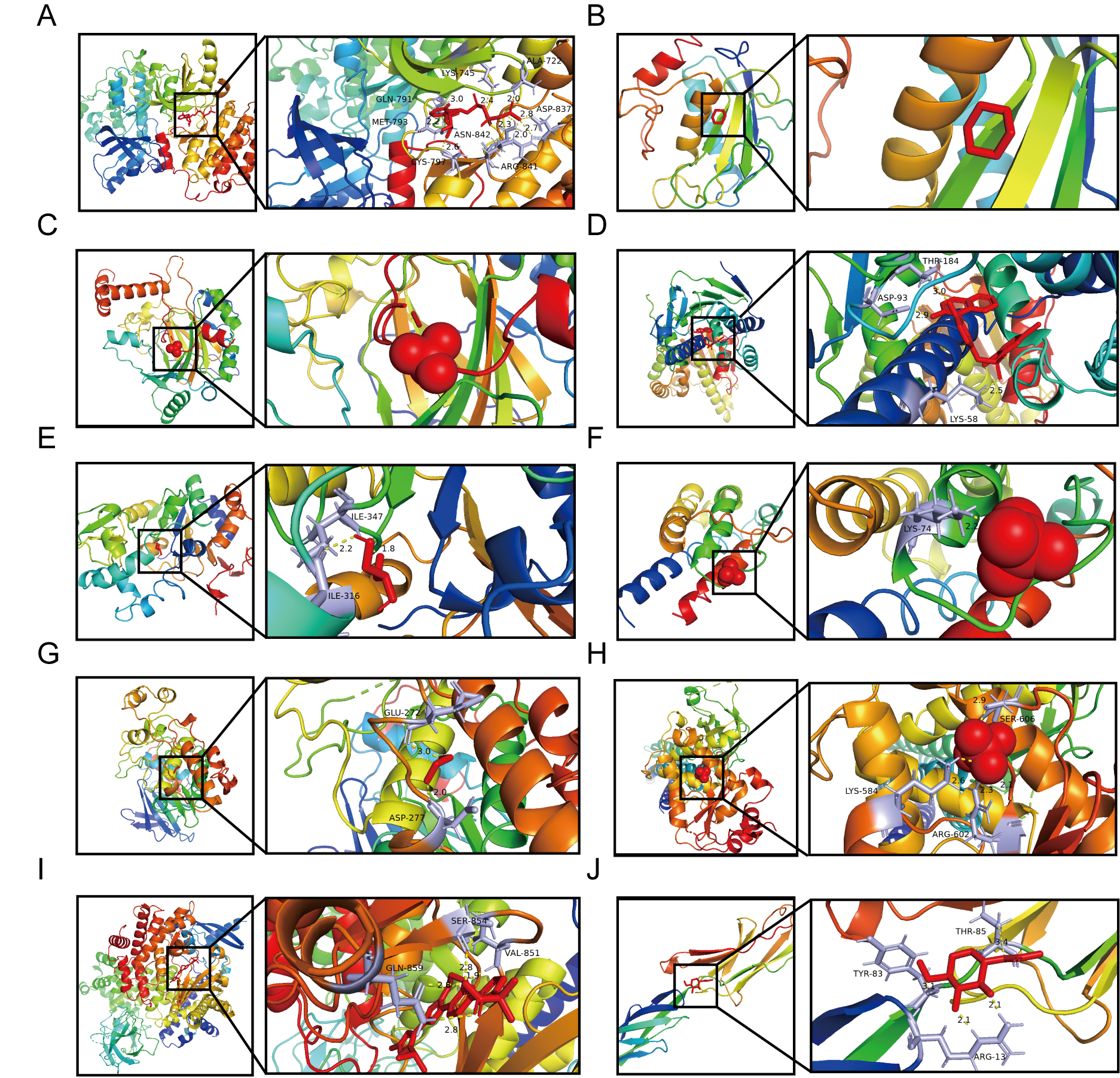

Supplement: Supplementary file 3 [file Image2.TIF]

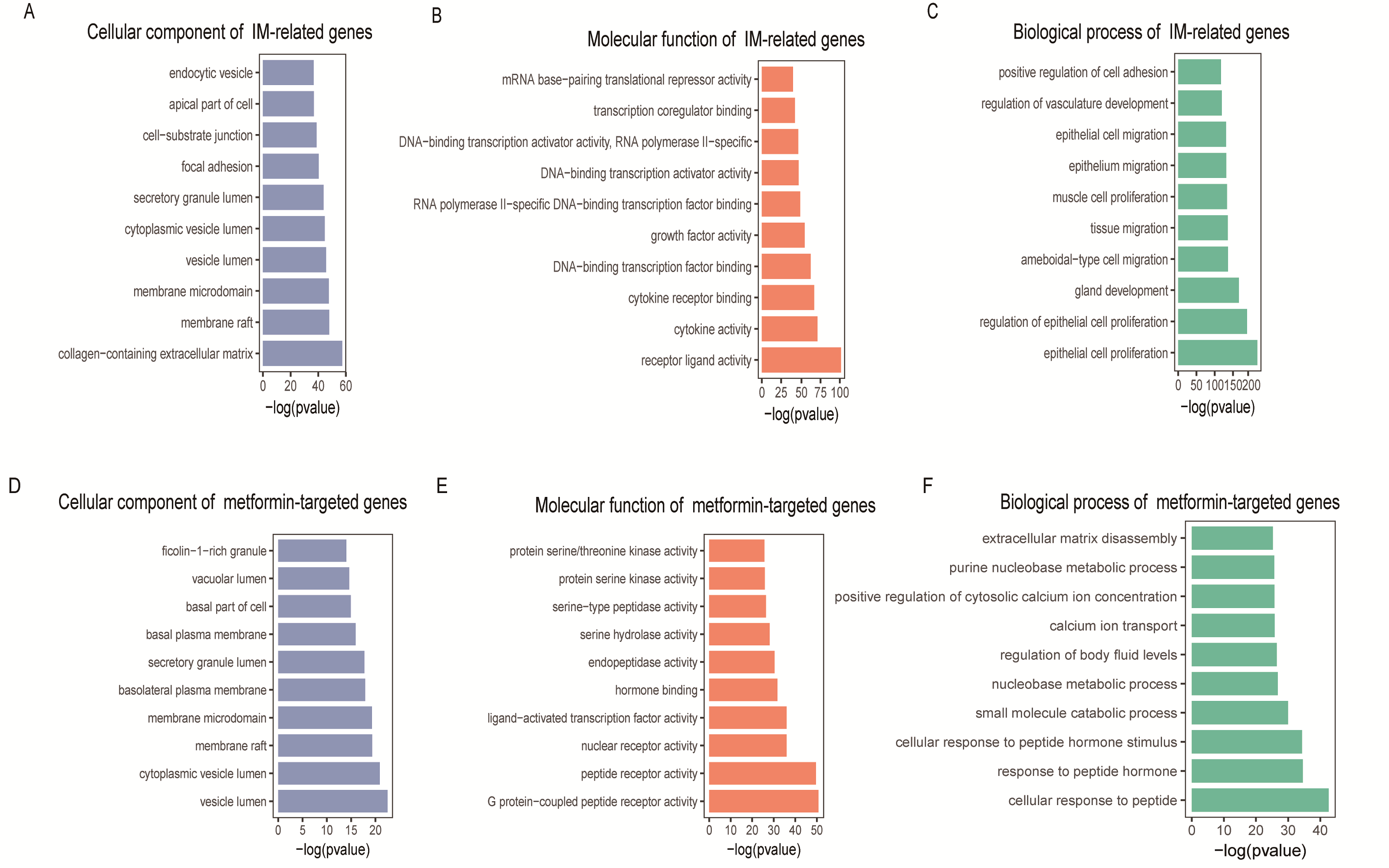

Supplement: Supplementary file 4 [file Image1.TIF]
